# Intravaginal immunization using the recombinant HIV-1 clade-C trimeric envelope glycoprotein CN54gp140 formulated within lyophilized solid dosage forms

**DOI:** 10.1016/j.vaccine.2011.04.023

**Published:** 2011-06-15

**Authors:** Louise Donnelly, Rhonda M. Curran, John S. Tregoning, Paul F. McKay, Tom Cole, Ryan J. Morrow, Vicky L. Kett, Gavin P. Andrews, A. David Woolfson, R. Karl Malcolm, Robin J. Shattock

**Affiliations:** a The School of Pharmacy, The Queen's University of Belfast, Medical Biology Centre, 97 Lisburn Road, Belfast BT9 7BL, Northern Ireland, UK; b Centre for Infection and Immunity, Clinical Sciences Division, St. George's University of London, London SW17 ORE, UK

**Keywords:** Intravaginal delivery, Mucosal vaccination, HIV-1, Lyophilized solid dosage formulations, DSC, differential scanning calorimetry, GNA, *Galanthus nivalis*, HEC, hydroxyethylcellulose, HPMC, hydroxypropylmethylcellulose, LSDFs, lyophilized solid dosage formulations, NaCMC, sodium carboxymethyl cellulose, PC, polycarbophil, PVP, polyvinylpyrollidone, RSVs, rheologically structured gel vehicles, SVF, simulated vaginal fluid

## Abstract

Vaccine-mediated prevention of primary HIV-1 infection at the heterosexual mucosal portal of entry may be facilitated by highly optimised formulations or drug delivery devices for intravaginal (i.vag) immunization. Previously we described hydroxyethylcellulose (HEC)-based rheologically structured gel vehicles (RSVs) for vaginal immunization of an HIV-1 vaccine candidate, a soluble recombinant trimeric HIV-1 clade-C envelope glycoprotein designated CN54gp140. Here we investigated the efficacy of lyophilized solid dosage formulations (LSDFs) for prolonging antigen stability and as i.vag delivery modalities. LSDFs were designed and developed that upon i.vag administration they would reconstitute with the imbibing of vaginal fluid to mucoadhesive, site-retentive semi-solids. Mice were immunized with lyophilized equivalents of (i) RSVs, (ii) modified versions of the RSVs more suited to lyophilization (sodium carboxymethyl cellulose (NaCMC)-based gels) and (iii) Carbopol^®^ gel, all containing CN54gp140. NaCMC-based LSDFs provided significantly enhanced antigen stability compared to aqueous-based RSVs. Rheological analysis indicated the NaCMC-based LSDFs would offer enhanced vaginal retention in woman compared to more conventional vaginal gel formulations. All LSDFs were well tolerated in the mouse model. Following i.vag administration, all LSDFs boosted systemic CN54gp140-specific antibody responses in sub-cutaneously primed mice. Induction of CN54gp140-specific antibody responses in the female genital tract was evident. Of all the LSDFs the fastest releasing which was lyophilized Carbopol^®^ gel elicited immune responses comparable to buffer instillation of antigen suggesting that rather than slower sustained release, initial high burst release from the LSDFs may suffice. The boosting of specific immune responses upon i.vag administration indicates that LSDFs are viable mucosal vaccine delivery modalities promoting antigen stability and facilitating intimate exposure of CN54gp140 to the mucosal-associated lymphoid tissue of the female genital tract.

## 1 Introduction

The HIV epidemic is fuelled predominantly by heterosexual transmission, notably so in sub-Saharan Africa where women are disproportionately infected particularly in the 15–24-year-old age range [1]. The exact mechanisms whereby HIV infects across the vaginal mucosal barrier are not fully elucidated [2], although studies with SIV indicate that HIV rapidly disseminates once across the vaginal epithelium [3]. As such, there is profound scientific rationale to pursue the development of female-controlled preventive strategies, principally involving the cervico-vaginal region, the predominant mucosal viral portal of entry in heterosexual transmission. To be fully effective, such a vaccine should provide sterilising immunity in the vaginal mucosal environment by inducing sustained robust protective immune effector function against diverse viral isolates.

How to achieve sustained immune effector function, particularly humoral immune effector function by way of neutralising antibody or rapid effective recall of immunological memory at mucosal surfaces is the subject of intense investigation. In addition, from a formulation/drug delivery perspective to ensure equity of access, particularly in the context of sub-Saharan Africa, such a vaccine should preferentially be inexpensive, safe, thermo-stable not requiring cold-chain storage and would facilitate female-controlled administration.

It is thought that the envelope spike is the only HIV-1 target available for neutralising antibodies [4]. As a result much emphasis has been placed on viral surface envelope glycoproteins as HIV-1 vaccine candidates. The efficacy of protein pharmaceuticals as vaccines depends upon maintaining storage stability as well as intended antigenicity following administration. Vaginally administered solubilised protein antigens are subject to leakage at the administration site, rapid enzymatic degradation, the influences of the menstrual cycle and inadequate exposure to the mucosal associated lymphoid tissue. There are a limited number of reports of vaginal immunization in women [5–11] and, with the exception of three studies [5–7] they have employed a known potent mucosal immunogen-cholera toxin subunit B that does not require the use of an adjuvant.

We previously reported on the design and development of well-tolerated mucoadhesive, syringeable, rheologically structured semi-solid vehicles (RSVs) for site-retentive vaginal administration of an HIV-1 vaccine candidate – a recombinant clade-C gp140 envelope protein (CN54gp140), in the rabbit model [12,13]. While the RSVs were a viable delivery modality for vaginal immunization as determined by the elicitation of vaccine-specific serum immunoglobulin (Ig) G, and vaccine-specific IgG and IgA in genital tract secretions, the vaccine was not stable within the aqueous-based preserved RSV formulations. The antigenicity of CN54gp140 altered over the course of prolonged storage and this was more pronounced the higher the storage temperature. In a related study, repeated vaginal exposure of CN54gp140, formulated within a Carbopol^®^ semi-solid, was well tolerated and induced systemic and mucosal IgG antibody responses in the rabbit model [14].

Herein we report the formulation and vaginal delivery of CN54gp140 within solid dosage forms; lyophilized equivalents of the Carbopol^®^, RSV and modified RSV semi-solid formulations. The innovative, robust, lyophilized solid dosage formulations (LSDFs) in this study were more conducive to CN54gp140 stability with the potential to offer improved patient acceptability for vaginal administration than the equivalent semi-solid formulations. In addition, the viability of the LSDFs as delivery modalities for vaginal immunization was demonstrated by the ability of the vaginally administered lyophilized formulations containing CN54gp140 to boost subcutaneously primed mice.

## 2 Materials and methods

### 2.1 Materials

Polyvinylpyrollidone (PVP) (Plasdone^®^ K-90, M_v_ 1.3 M) and Polycarbophil (PC) (Noveon^®^ AA1, divinyl crosslinked polyacrylic acid) were kindly donated by International Speciality Products (Ohio, USA) and Noveon Pharma GmbH & Co KG (Raubling, Germany), respectively. HEC (Natrosol 250 HHX and 250 G) and sodium NaCMC (Blanose^®^ 7LF, 7MF, and 7HF) were also kindly donated by Aqualon (Warrington, UK). GMP manufactured Carbopol^®^ 974P gel, formulation #2449 was kindly donated by Particle Sciences (Bethlehem, PA, USA). *Galanthus nivalis* (GNA) was obtained from Vector Laboratories (Peterborough, England). 3,3′,5,5′-Tetramethylbenzidine peroxidase substrate (TMB/E) was obtained from Cygnus Technologies Inc. (North Carolina, USA). CN54gp140 (gp120 plus the ectodomain of gp41) was encoded by the CN54gp140REKE HIV-1 envelope gene cassette derived from the clade-C/B′ HIV-1 molecular clone p97CN54 of Chinese origin developed by Wolf and Wagner, University of Regensburg, Germany [15,16]. CN54gp140 was produced as a recombinant product in CHO cells by S. Jeffs, Imperial College, London, and manufactured to GMP specification by Polymun Scientific (Vienna, Austria) who also donated the HIV-1 gp41 specific monoclonal antibody 5F3 (HuMab 5F3). Sodium hydroxide, phosphate buffered saline containing Tween 20 (PBS-T), sterile-filtered porcine serum and goat anti-human horseradish peroxidase (HRP)-conjugated IgG were purchased from Sigma–Aldrich (Poole, Dorset, UK). Goat anti-mouse HRP-conjugated IgA and biotinylated goat anti-mouse IgA were obtained from AbD Serotec (UK). HRP-conjugated streptavidin was purchased from R&D Systems (MN, USA). 25X protease inhibitor cocktail was obtained from Roche (Hertfordshire, UK). Reactibind 96 well microplates were obtained from Perbio Science (Northumberland, England). Nunc Maxisorp 96 well microplates were obtained from Nalge Nunc International (Rochester, NY). Nalgene tubing (PVC, 3 mm internal diameter, 5 mm outer diameter, 1 mm Wall) was purchased from VWR International Ltd. (Dublin, Ireland) and blister packs were kindly supplied by Almac (Craigavon, UK) and Warner Chilcott (Larne, UK). Ultra-pure water was obtained using an Elga Purelab Maxima system. Six to 8-week old female BALB/c mice were obtained from Harlan Ltd., UK. All *in vivo* procedures were carried out in compliance with the United Kingdom Animal (Scientific Procedures) Act 1986 and associated Codes of Practice for the Housing and Care of Animals.

### 2.2 Preparation of semi-solids

#### 2.2.1 Hydroxyethylcellulose (HEC) based semi-solids

Preparation of the HEC based RSV formulations has been described previously [13]. Briefly, a HiVac^®^ Bowl (Summit Medical Ltd., Gloucestershire, UK) was used to facilitate mixing under vacuum following the stepwise addition of components. Poylcarbophil (PC) (3% w/w) was first added to the bowl containing deionised water and sodium hydroxide prior to the addition of HEC (3 or 5% w/w) followed by polyvinylpyrollidone (PVP) (4% w/w).

#### 2.2.2 Sodium carboxymethyl cellulose (NaCMC) based semi-solids

PC (3% w/w) was added to the vortex produced in a metal beaker by rapid stirring (at 500 rev min^−1^) of deionised water and the required amount of NaOH to reach pH 6 using a Heidolph mechanical stirrer. Following complete dissolution of the mucoadhesive component, NaCMC (3, 5 or 10% w/w) and PVP (4% w/w) were added stepwise following attainment of homogeneity. The gels were transferred to sterile centrifuge tubes, gently centrifuged and stored for 24 h (ambient temperature) prior to analysis.

### 2.3 Flow analysis of semi-solids pre-lyophilization under continuous shear

Flow rheometry was conducted using an AR2000 rheometer (T.A. Instruments, Surrey, England) at 25 ± 0.1 °C using a 6 cm diameter parallel plate geometry (selected according to formulation consistency) and a gap of 1000 μm, as previously reported [12]. Flow curves (plots of viscosity versus shear rate) were examined in the range of 0.1–100 s^−1^.

### 2.4 Preparation of CN54gp140 loaded semi-solids

#### 2.4.1 For lyophilized solid dosage tablet formation

NaCMC semi-solid (2.8 g) was weighed into a 5 ml syringe barrel. The semi-solid loaded syringe barrel was attached to a second syringe via a 1.5 cm length of Nalgene tubing. CN54gp140 (200 μl at 530 μg/ml) was added to the semi-solid containing syringe barrel via pipette and the plunger replaced. Uniform distribution of CN54gp140 throughout the semi-solid formulation was achieved by carrying out 40 passes of the syringe barrel contents from one syringe to the other (method previously validated [13]).

#### 2.4.2 For lyophilized solid dosage rod formation for mouse immunization studies

Semi-solids (HEC- and NaCMC-based) (0.36 g) were weighed into a speed mixing pot prior to the addition of CN54gp140 (180 μl at 3.5 mg/ml). 2 Spin cycles at 3300 rpm for 30 s were carried out to provide uniform antigen distribution throughout the semi-solid formulations. The same lyophilization protocol was adopted for each formulation.

### 2.5 Differential scanning calorimetry (DSC) of semi-solids pre-lyophilization

To optimise the lyophilization protocols, the glass transition temperatures of the selected and cooled semi-solid formulations were investigated by DSC using hermetic pans (DSC Q100, TA Instruments, Surrey, UK). Following cooling to −60 °C and holding isothermally for 5 min, the samples were heated at 2–40 °C using a modulated procedure (±0.4 °C every 0.5 s).

### 2.6 Lyophilization

Prior to lyophilization, semi-solid formulations were dispensed into suitable blister packs using a TS250 Digital Timed Dispenser (Adhesive Dispensing Ltd., Buckinghamshire, UK) for tablet formation or alternatively extruded into nalgene tubing with the use of a 5 ml syringe for rod formation. Lyophilization was carried out using the Advantage Freeze Dryer (VirTis, Gardiner, NY, USA). Blister packs/tubing were placed on the shelf and a 4 h thermal treatment step was carried out at −28 °C. This temperature was maintained for a further 2 h while the chamber pressure was reduced to 100 mTorr. Primary drying commenced with a 4 h hold under these conditions followed by a 1 h ramp to and 2 h hold at −20 °C. The temperature was further ramped to 0 °C over 2 h then held for 2 h at 500 mTorr followed by a 2 h ramp to 20 °C. Secondary drying was then performed at 27 °C for 4 h at a reduced pressure of 50 mTorr. Following lyophilization samples were transferred into individual sterile universal tubes.

### 2.7 Friability studies

Each lyophilized solid dosage tablet formulation tested (*n* = 5) was weighed and transferred into the test drum of a Copley Scientific friability tester (25 rpm, 4 min), during which they are subjected to the rolling movement around the drum which has a curved aperture allowing the formulations to rise and then fall over a distance of ∼16 cm. The dosage forms were then expelled, reweighed and any decrease in weight recorded.

### 2.8 *In vitro* dilution of lyophilized solid dosage tablet formulations in simulated vaginal fluid (SVF)

SVF was prepared as previously described [17]. NaCl (3.51 g), KOH (1.40 g), Ca(OH)_2_ (0.222 g), bovine serum albumin (BSA) (0.018 g), lactic acid (2 g), acetic acid (1 g), glycerol (0.16 g), urea (0.4 g) and glucose (5 g) were dissolved in 1 L of deionised water, followed by adjustment to pH 4.2 with HCl. Solid dosage tablet formulations were diluted and thoroughly mixed with a defined volume of SVF (1 ml) and the dynamic rheological properties analyzed. Oscillatory rheometry was conducted within the linear viscoelastic region over a frequency range from 0.1 to 10 Hz as described elsewhere [12]. The dilution ratio was chosen on the basis of that normally encountered in the vagina following insertion of the delivery vehicle [17].

### 2.9 ELISA for the quantification of CN54gp140

A heterogeneous indirect sandwich ELISA was optimised for quantification of CN54gp140 in PBST (linear concentration range 0.003–0.05 μg/ml, *R*^2^ > 0.999). Wells were incubated with 50 μl/well of GNA at 10 μg/ml in deionised water (5 h at 37 °C). The wells were washed (5× 300 μl PBS-T) and blocked for 1 h at 37 °C with PBST containing 5% porcine serum (PBS-T-serum). Standards, samples and controls were prepared in PBS-T (*n* = 4), and incubated overnight at ambient temperature. The wells were washed and incubated with 50 μl/well HuMab 5F3 (1 μg/ml in PBS-T-serum) for 2 h at 37 °C. Following washing, bound antibody was detected using 50 μl/well goat anti-human IgG peroxidase conjugate diluted 1:5 K in PBS-T-serum and incubated for 1 h at 37 °C. After washing, the wells were incubated with 100 μl TMB/E for 5 min. The reaction was terminated by the addition of 50 μl of 2.5 M H_2_SO_4_. Plates were read immediately at *A*_450_.

### 2.10 *In vitro* release of CN54gp140 from lyophilized solid dosage tablet or rod formulations

Each CN54gp140 containing lyophilized tablet (*n* = 4) was placed in a sterile polypropylene pot containing release media (PBS-T, 30 ml) prior to housing in an orbital incubated shaker (Sanyo Gallenkamp, 100 rpm) maintained at 37 °C. At predetermined time intervals the release medium was sampled (3 ml) and replaced with fresh pre-warmed dissolution media. Samples were diluted in PBS-T for concentration analysis by ELISA. For rods dissolution volume was 20 ml and sample volume was 2 ml. Dissolution volumes were selected to maintain sink conditions.

### 2.11 CN54gp140 stability assessment within lyophilized solid dosage tablet formulations

Stability assessment was carried out in a similar fashion to the described release protocol. Following complete dissolution of the CN54gp140 containing lyophilized solid dosage tablets in PBS-T (30 ml) a sample was taken and diluted in PBS-T for concentration analysis by ELISA.

### 2.12 Mouse immunogenicity study

Animals were assigned to experimental groups where *n* = 5 (Table 1). Mice received a subcutaneous (s.c.) prime (Day 0) then an intra-vaginal (i.vag.) boost three times at 21-day intervals (Days 21, 42, 63) with vaginally administered rod formulations (Table 1). Mice were lightly anesthetised and the rod formulations were inserted into the vagina using a positive displacement pipette (Gilson Microman – 100 μl maximum volume) and a tip with the end cut off and filed down to smoothness. To thin the vaginal epithelium and improve protein uptake, mice were treated subcutaneously with 2 mg of depoprovera (in 50 μl PBS) 5 days prior to the first and third vaginal immunization. Blood samples were taken from the tail vein of mice on Days 20, 41, 62, and 83 and by cardiac puncture on Day 120. Blood samples were centrifuged following clotting for collection of sera. Vaginal lavages were conducted on Day 83. Vaginal lavages were collected and pooled by flushing the vaginal lumen three times with a 25 μl volume of PBS using a positive displacement pipette. 5 μl of 25X protease inhibitor cocktail was added to the vaginal eluates, which were incubated on ice for 30 min prior to centrifugation to remove the mucus/cellular pellet. All samples were stored at −80 °C until analysis.

### 2.13 CN54gp140-specific antibody ELISAs

Binding antibodies against CN54gp140 in vaginal lavage and serum samples were measured a quantitative ELISA. 96-Well plates were coated with CN54gp140 and blocked with 1% BSA as before. IgG or IgA standards were used on each plate to quantify the CN54gp140 specific antibodies. Experimental samples were diluted 1:100, 1:1000 and 1:10,000 (sera) or 1:10 and 1:50 (lavage) to ensure the absorbance reading measured fell within the linear range of the standard curve. Bound IgG was detected by incubation for 1 h at 37 °C with HRP-conjugated goat anti-mouse IgG, bound IgA was detected using biotinylated anti-mouse IgA and followed by Streptavidin-HRP. Plates were washed and developed with 50 μl TMB/E substrate and the reaction was terminated by the addition of 50 μl of 2 M H_2_SO_4_ and read at *A*_450_. Vaginal lavage values were normalised against the total IgA or IgG measured in the same sample.

## 3 Results

### 3.1 Preparation of semi-solids

Semi-solids (Table 2) were prepared using either an overhead stirrer or HiVac^®^ bowl, the choice of which was dependent upon the viscosity of the systems being prepared. The lower viscosity of the NaCMC-based semi-solids permitted use of the overhead stirrer and although some degree of aeration occurred this was readily removed upon gentle centrifugation (min). It had previously been determined that overhead stirring was not suitable for preparation of the HEC-based semi-solids due to the high rate of shear required to achieve uniform mixing, excessive aeration and the potential for high shearing stresses to trigger mechanical breakdown of the polymeric components. To overcome this, mixing was carried out under vacuum with the use of the HiVac^®^ mixing bowl.

### 3.2 Flow analysis of semi-solids pre-lyophilization under continuous shear

**Fig. 1:**
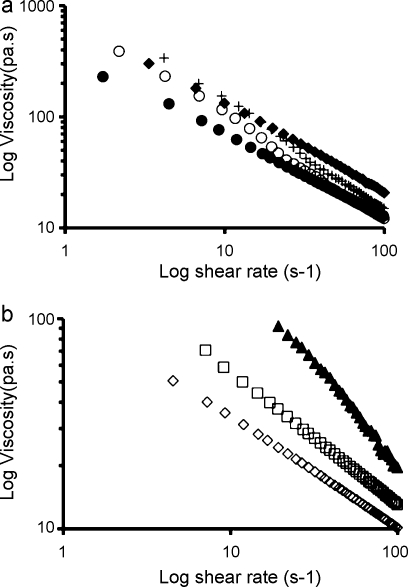
Mean log viscosity versus log shear rate (*n* = 4) of semi-solid formulations at 25 °C prepared from [a] (●) PC3HEC250G1, (○) PC3HEC250HHX1, (+) PC3HEC205HHX5, (♦) PC3HEC250HHX1PVP4, and [b] (▴) PC3Blanose7LF10PVP4, (□) PC3Blanose7LF5PVP4, (♢) PC3Blanose7LF3PVP4. Standard error bars have been omitted for clarity – coefficient of error<5% throughout.

Following dispensing trials a number of semi-solid formulations were selected for rheological flow analysis. The influence of shear rate on the shear viscosity of the selected HEC- and NaCMC-based semi-solids is shown in Fig. 1a and b, respectively. Flow analysis showed that all the semi-solid formulations were pseudoplastic in nature in that they displayed decreasing shear viscosity with increasing shear rate. The power law function was used to determine flow consistency (*κ*) of the materials understudy (at 1 s^−1^) (Table 2).

### 3.3 DSC analysis of semi-solids pre-lyophilization

On the basis of rheological analysis and dispensing trials, determined by viscosity and ability to settle into blister pack wells, formulations containing Blanose 7LF were chosen for lyophilization. For all semi-solid formulations in the absence and presence of CN54gp140, the glass transition temperature was identified between −21 and −22 °C.

### 3.4 Lyophilization

**Fig. 2:**
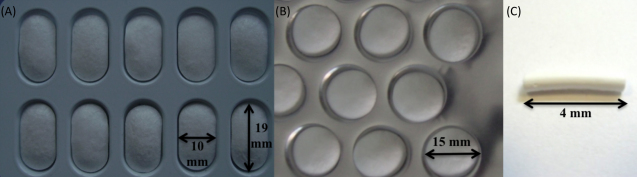
Solid dosage formulations following lyophilization of NaCMC-based semi-solids. (a) LSDFs suitably sized for i.vag administration using conventional human vaginal applicators, (b) alternative LSDFs using round tablet blister pack wells for dispensing, and (c) LSDFs used for murine i.vag administration in the mouse immunogenicity study.

Three solid dosage forms with different dimensions were prepared (Fig. 2a–c). LSDFs containing 10% Blanose 7LF were inconsistent in structure whereas those containing lower levels of Blanose 7LF provided uniform units suitable for further investigation.

### 3.5 Friability studies

Following friability testing no lyophilized solid dosage formulation tested (both those shown in Fig. 2a and b) was subject to fracture or exterior damage. No loss of weight occurred whereas slight increases in weight were detected (<8%).

### 3.6 SVF dilution studies

Following reconstitution of the LSDFs designed for i.vag administration (Fig. 2a) in SVF (1 tablet per 1 ml) oscillatory (dynamic) analysis (a measure of consistency) was performed on the resulting semi-solid structure at 37 °C and compared to the original equivalent semi-solid formulations pre-lyophilization (Table 2).

### 3.7 *In vitro* release of CN54gp140 from lyophilized solid dosage formulations

**Fig. 3:**
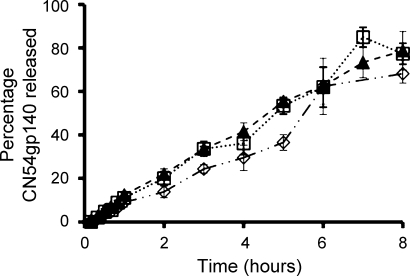
Mean (±1 SD) *in vitro* percentage cumulative release of CN54gp140 (*n* = 4) from lyophilized NaCMC-based solid dosage tablet formulations (□) lyo-PC3Blanose7LF10PVP4, (▴) lyo-PC3Blanose7LF5PVP4, (♢) lyoPC3Blanose7LF3PVP4.

The percentage cumulative release of CN54gp140 from solid dosage formulations (formulation type – Fig. 2b) containing Blanose 7LF at 3, 5 and 10% is shown in Fig. 3. Release profiles of CN54gp140 were similar, displaying a continuous release of antigen with maximum CN54gp140 detectable (Tmax) in the dissolution media after a 7–8 h period (Table 3).

**Fig. 4:**
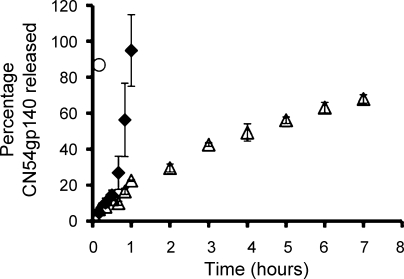
Mean (±1 SD) *in vitro* percentage cumulative release of CN54gp140 (*n* = 4) from lyophilized rods (formulation type – Fig. 2c) going forward to the mouse immunogenicity study (○) lyo-Carbopol^®^, (♦) lyo-PC3Blanose7LF3PVP4 and (▵) lyo-PC3HEC250HHX5PVP4.

The percentage cumulative release of CN54gp140 from solid dosage formulations (formulation type – Fig. 2c) lyo-PC3HEC250HHX5PVP4, lyo-PC3Blanose7LF3PVP4 and lyo-Carbopol^®^ going forward to the mouse immunogenicity study are shown in Fig. 4.

### 3.8 CN54gp140 stability assessment within lyophilized solid dosage tablet formulations

**Fig. 5:**
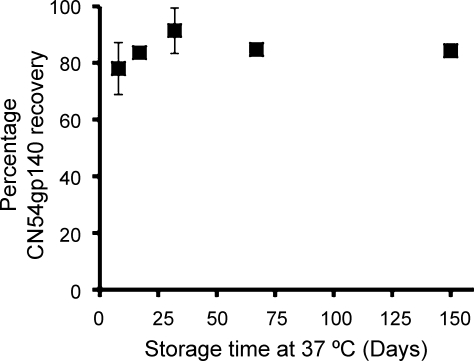
Mean (±1 SD) recovery of CN54gp140 (*n* = 4) from lyo-PC3Blanose7LF3PVP4 solid dosage tablets (formulation type – Fig. 2b) following prolonged storage at 37 °C.

Stability of CN54gp140 within the lyophilized solid dosage tablet formulation (Formulation type – Fig. 2b) lyo-PC3Blanose7LF3PVP4 was assessed following prolonged storage at 37 °C. The percentage recovery of CN54gp140 is shown in Fig. 5. No loss in recoverable CN54gp140 (>70%) was experienced over the duration of the study.

### 3.9 Mouse immunogenicity study

**Fig. 6:**
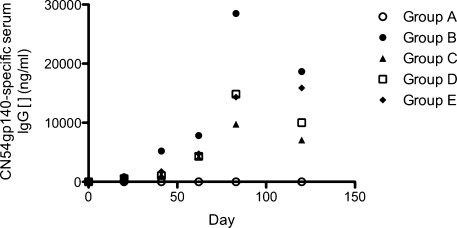
CN54gp140-specific serum IgG time-course (*n* = 5). (○) Group A, (●) Group B, (▴) Group C, (□) Group D and (♦) Group E. Error bars omitted for clarity.

**Fig. 7:**
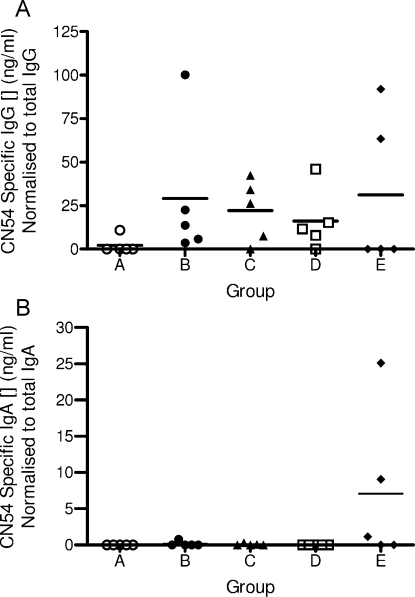
CN54gp140-specific IgG (a) and IgA (b) responses detected in vaginal lavage samples (Day 83). Values are normalised to total IgG or IgA measured in the same sample. (○) Group A, (●) Group B, (▴) Group C, (□) Group D (♦) and Group E. Error bars represent ±1 SD.

All pre-treatment serum samples and those from the control naïve experimental Group A at every time point tested negative for CN54gp140-specific IgG and IgA antibody (Fig. 6). With the exception of one apparent responder in Group D, CN54gp140-specific IgA responses were neglible. Group B exhibited a significantly enhanced CN54gp140-specific serum IgG response on Days 41 and 83 against other groups and compared to the naïve control Group A (*P* < 0.01; Dunnet Multiple Comparisons test). Furthermore, Groups B and E had significant CN54gp140-specific serum IgG responses by Day 120, against other groups and compared to the naïve control Group A (*P* < 0.01 and *P* < 0.05, respectively; Dunnet Multiple Comparisons test). Interestingly, Group E maintained CN54gp140-specific IgG antibody responses between Days 83 and 120 while in all other the responding groups the antibody levels had waned as expected with the final vaccination have been given at Day 63 (Fig. 6). To determine mucosal immune responses, CN54gp140-specific IgG (Fig. 7a) and IgA (Fig. 7b) were quantified in vaginal lavage. CN54 specific IgG was detectable in the vaginal lavage of immunized mice, IgA was only detectable in the carbopol group.

## 4 Discussion

To the best of our knowledge, this article is the first example of i.vag immunization employing LSDFs derived from semi-solids. Previously soluble recombinant HIV-1 gp140 has been shown to be immunogenic in the absence of mucosal adjuvant, upon i.vag immunization and formulated within semi-solids [13,14]. This is the first demonstration that soluble recombinant HIV-1 gp140 is immunogenic in the absence of mucosal adjuvant, upon i.vag immunization, and formulated within LSDFs. Moreover, the formulations were well tolerated in the murine model.

In general, semi-solid dosage forms are currently the most common dosage form used for i.vag delivery [18]. They have many desirable attributes that make them suitable for vaginal delivery but are also associated with messiness and poor retention. Previously we developed highly viscous, mucoadhesive gel systems, developed for site-retentive application of CN54gp140 to the vagina [13]. Although the GMP manufactured CN54gp140 has proven to be exceptionally stable in simple buffer solutions (D. Katinger – personal communication), stability was severely compromised when formulated within the aqueous-based RSVs. So although both the RSVs and a considerably less viscous Carbopol^®^ semi-solid formulation [13,14] have proven to be viable delivery modalities for i.vag immunization with CN54gp140, from a practical perspective such aqueous-based semi-solid formulations requiring labour intensive bed-side mixing to overcome instability concerns are neither suitable for the clinic or field.

Lyophilization is routinely used for the preparation of stable pharmaceutical products. In the present study lyophilization of semi-solids was explored with the intention of developing LSDFs for i.vag immunization that were conducive to antigen stability. Desirable attributes of the resulting LSDFs included that they would provide rapid stabilisation of antigen, long-term product stability (avoiding cold-chain storage) and ease of reconstitution upon i.vag administration. Upon administration these formulations were predicted to reconstitute to semi-solids by the imbibing of vaginal fluid, permitting intimate contact of the vaccine candidate with the vaginal epithelium. Upon reconstitution the formulations would retain the intended beneficial attributes of the original semi-solid formulations, including mucoadhesiveness and in the case of the lyophilized RSVs enhanced viscoelasticity, thus enhanced retention compared with more conventional vaginal semi-solid formulations, including Carbopol^®^.

To enable preparation of the LSDFs, equivalent to their respective semi-solid formulations but with defined dimensions (suitably translational to the human clinic), semi-solids were dispensed into blister packs and subsequently lyophilized. Due to their high viscosity and resistance to deformation the RSVs described previously [12,13] were not suitable for dispensing, as they were resistant to settling within wells. The RSV semi-solid formulation (PC3HEC250HHX5PVP4) [12] underwent modification to reduce viscosity thus facilitating lyophilization in blister packs, determined visually through dispensing trials and by rheological flow analysis (manifested as a reduction in viscosity). Modifications trialled included a reduction in the HEC250HHX content from 5% to 1%, omission of the PVP component, and omission of the PVP component plus substitution of the HEC250HHX polymer component with HEC250G, a grade exhibiting lower Brookfield viscosity (400 mPa s compared to 15,000 mPa s). Rheological flow analysis, used as an aid for the optimisation of processing parameters such as dispensing, in addition to predicting the way in which a material will behave upon storage and end-user application, demonstrated the pseudoplastic nature of all the modified RSVs. Such shear thinning behaviour was a desirable attribute to facilitate expulsion of the semi-solids from the dispensing tubes and to ensure adequate settling into the blister pack wells. Omission of the PVP component had no significant effect on consistency (determined using power law) whereas reduction of the HEC250HHX content resulted in a drop in consistency from 3194 ± 177 Pa s^*n*^ [12]. Substitution with HEC250G in combination with PVP omission also resulted in a drop in consistency to 399 ± 14 Pa s^*n*^. However, dispensing trials demonstrated that the HEC-based semisolids did not exhibit sufficient flow properties to settle uniformly into the blister pack wells.

To overcome this, the HEC component of the original RSV formulations was substituted with NaCMC. NaCMC-based semi-solid formulations were subsequently evaluated, and the original RSV formulation was not pursued to lyophilization as uniform dosage forms suitable for human administration were not attainable. Pharmaceutical grades of Blanose were investigated with high (7HF), medium (7MF) and low (7LF) molecular weights (MW). The Brookfield viscosity of Blanose decreases down through the grades – 7HF (20,000 mPa s), 7MF (600 mPa s) and 7LF (40 mPa s). As a result when combined with PVP and PC the consistency of the semi-solid formulations decreased with decreasing MW of the Blanose component. The higher viscosity arising from the inclusion of the 7HF grade is likely due to the increased number of physical entanglements that the larger molecular weight component may form, which in turn may lead to the increased resistance to deformation observed in the form of resistance to settling into the blister pack wells. In contrast to this, inclusion of the 7LF grade resulted in the formulation of semi-solids that could be adequately dispensed and subsequently settled into the blister pack wells. As a result the optimised LSDFs described contain Blanose 7LF as part of their overall polymer component.

To avoid collapse of the formulations during lyophilization DSC analysis was conducted to optimise the lyophilization protocols. Primary drying was maintained below the glass transition temperatures of the semi-solids at −28 °C to overcome inefficiency of thermal transfer between the shelf and dispensed semi-solids and to ensure immobilisation of the polymeric chains thus preserving structure. Friability testing indicated that the solid-dosage formulations would withstand the rigors of transport and handling. The slight increases in batch weight were attributed to water uptake upon re-exposure of the dehydrated formulations to normal atmospheric conditions.

As anticipated oscillatory analysis confirmed a decrease in consistency of the semi-solid formulations created upon reconstitution of the LSDFs with SVF compared to the equivalent semi-solids pre-lyophilization. This was attributed to the lower pH and lower ratio of solid polymer component to solution of the reconstituted systems. Although, compared to the original RSV semi-solid formulations the viscosity of the Blanose containing formulations prior to lyophilization and following reconstitution in SVF was considerably reduced, the reconstituted formulations (modelling the *in vivo* scenario) retained consistencies greater than those of commercially available PC based formulations [12] prior to i.vag administration. Importantly, based on this observation the LSDFs were anticipated to offer enhanced vaginal retention compared to more conventional gels such as Carbopol^®^ which would be subject to further reductions in viscosity upon i.vag administration due to the imbibing of vaginal fluid, increases in temperature and exposure to lower pH.

*In vitro* release of CN54gp140 from the LSDFs containing Blanose 7LF was sustained over an 8 h period at which point the tablets had undergone complete dissolution. As anticipated due to changes in viscosity the LSDFs containing Blanose 7LF release approximately two-fold faster upon reconstitution (modelling the *in vivo* scenario) than the highly viscous RSVs (expulsed into dissolution medium to model *in vivo* smearing) [13]. The percentage loading of Blanose 7LF did not influence *in vitro* release. As a result one lyophilized formulation lyo-PC3Blanose7LF3PVP4 was progressed to stability and immunogenicity analysis. The degree of matrix associated dampening varies with each formulation type and over the course of a dissolution study using the specified ELISA. Therefore the concentration of CN54gp140 was determined against a CN54gp140 in PBS-T calibration curve and matrix associated dampening was not corrected for. As a result recovery of CN54gp140 as determined by ELISA was not expected to reach 100%. Importantly antigenicity/recovery was retained at greater than 70% for at least 5 months when CN54gp140 was formulated within lyo-PC3Blanose7LF3PVP4 indicating that lyophilization significantly enhanced long-term stability under accelerated storage conditions. Comparatively, recovery had dropped in the aqueous-based RSVs from 77% to 21% by Day 9 at 37 °C [13]. PVP, one of the polymer components of the LSDFs is reported to be a cryoprotectant [19,20], which may have been a contributing factor.

Comparative *in vitro* release studies were also conducted on the LSDFs intended for the mouse immunogenicity study (Fig. 2c). The rationale for comparing the optimised Blanose 7LF containing LSDF to lyophilized equivalents of the original RSV and of Carbopol^®^ in the mouse immunogenicity study was that the selected formulations present three very different rates of release. The RSV and Carbopol^®^ gel can be lyophilized in rod format only. As previously discussed the RSV is not suitable for lyophilization within blister pack wells and the lyophilized equivalent of Carbopol^®^ gel is spongy with inadequate rigidity for i.vag administration. Due to their small size the *in vitro* release profiles of the lyophilized rods were of limited value and were merely designed to be demonstrative that due to the nature of the formulation components these rods release antigen at varying rates *in vitro* as was the case with the equivalent formulations of larger size. As anticipated the lyophilized version of Carbopol^®^ gel (lyo-Carbopol^®^) exhibited rapid release whereas the lyophilized version of the highly viscous unmodified RSV (lyo-PC3HEC250HHX5PVP4) had a much more sustained release profile. Comparative release profiles of the larger equivalent formulations designed for non-human primate (NHP) or human administration present more distinguishable release profiles further separating the quick release formulations from the more sustained release formulations. Inevitably formulation size is largely dictated by the constraints of animal models. How influential this is to enabling adequate comparison of formulations that are designed to release at varying rates *in vivo* is yet to be determined.

HIV envelope proteins are notoriously poorly immunogenic. Contrary to our previously conducted rabbit experiments [14] prior experiments in mice have indicated that i.vag as a sole route of administration for CN54gp140 alone does not elicit detectable immune responses (unpublished data). As a result we selected a heterologous prime-boost regimen, increasingly prevalent in HIV vaccine research [21]. Remarkably, all topically administered i.vag formulations boosted sub-cutaneously primed mice, importantly in the absence of adjuvant. Of the responses detected locally within the vagina we cannot rule out, as has been reported in HIV infection [22], that serum transudation contributed. Nevertheless, the LSDF inserts have been shown to be a viable delivery modality for i.vag immunization. With respect to immunogenicity the study data indicated that in the case of the mouse model the LSDFs were not offering any additional benefits over i.vag administration of CN54gp140 formulated within PBS buffer alone. Perhaps with the exception of lyophilized Carbopol^®^ that may be prolonging or augmenting CN54gp140-specific systemic humoral effector immune responses. The formulation (lyo-PC3HEC250HHX5PVP4) with the slowest release induces the lowest response, whereas the formulation (lyo-Carbopol^®^) with the fastest release closest to the PBS alone scenario marginally prolongs or augments the response. How translational this may be to other animal models, in particular NHPs and more importantly to humans is yet to be determined but this may be indicative that sustained release is not required rather an initial high burst release may suffice. The perceived benefits such as enhanced retention that drive such formulation development with respect to improving immune responses may not be wholly realised due to the size restrictions of the murine vaginal lumen.

However although the LSDFs did not augment immune responses in comparison to those following administration of antigen in PBS alone the problems associated with human i.vag administration of vaccines in simple buffer solutions are not to be underestimated. As such the LSDFs that elicited comparable immune responses to those of the PBS group have the potential to provide additional attributes for vaginal mucosal vaccine delivery in humans. LSDFs can be self-administered with relative ease using conventional solid dosage vaginal applicators, compared to the instillation of buffers and to the administration of semi-solids, thus promoting higher acceptability and enhanced user compliance. The stability advantages have the potential to eliminate the requirement for cold-chain storage, and the reduction in weight associated with the removal of water could reduce constraints on distribution including expense.

## 5 Conclusion

This study has described the design and development of LSDFs derived from the lyophilization of mucoadhesive semi-solid dosage forms. CN54gp140 was formulated within the LSDFs for i.vag administration. Upon application the LSDFs boosted s.c. primed mice indicating that the LSDFs reconsituted *in vivo* with the imbibing of vaginal fluid, resulting in intimate exposure of CN54gp140 with the mucosal-associated lymphoid tissue of the female genital tract. The LSDFs were conducive to long-term antigen storage stability. To the best of our knowledge this is the first description of lyophilized solid dosage forms as vaginal mucosal vaccine delivery modalities.

## Figures and Tables

**Table 1:** Assignment of animals to experimental groups.

Experimental group	s.c. Prime Day 0	i.vag boost (Day 21, 42, 63)
A	50 μl PBS	50 μl PBS
B	10 μg CN54gp140 in 50 μl PBS	10 μg CN54gp140 in 50 μl PBS
C	10 μg CN54gp140 in 50 μl PBS	10 μg CN54gp140 in a lyophilized equivalent of PC3HEC250HHX5PVP4 (5% HEC-based RSV) semi-solid (13)
D	10 μg CN54gp140 in 50 μl PBS	10 μg CN54gp140 in a lyophilized equivalent of PC3Blanose7LF3PVP4 semi-solid
E	10 μg CN54gp140 in 50 μl PBS	10 μg CN54gp140 in a lyophilized equivalent of Carbopol^®^ semi-solid

A group receiving a single s.c. injection of CN54gp140 was omitted as previous experiments have indicated this results in negligible titres (unpublished data).

**Table 2:** Polymer composition of semi-solid formulations and flow rheological properties.

Concentration of semi-solid components (% w/w)							Flow rheology
PC	PVP	HEC 250		NaCMC: Blanose			Viscosity/consistency (*κ*) (Pa s^*n*^) (25 °C)
		HHX	G	7HF	7MF	7LF	
3	4	1					857 ± 29
3		1					812 ± 49
3			1				399 ± 14
3	4			5			1210 ± 119
3	4			3			687 ± 15
3	4				5		837 ± 63
3	4				3		439 ± 8
3	4					10	978 ± 40
3	4					5	235 ± 7
3	4					3	121 ± 3

**Table 3:** Polymer composition of NaCMC-based semi-solids selected for lyophilization, oscillatory rheological properties pre- and post-lyophilization and *in vitro* release data post-lyophilization.

Conc. of semi-solid components (%w/w)			Oscillatory rheology data: Consistency			*In vitro* release data
			(25 °C)	(37 °C)	(37 °C)	
PC	PVP	NaCMC: Blanose	Semi-solid pre-lyophilization	Semi-solid pre-lyophilization	SVF reconstituted	% release
		7LF	(Pa s) *x* ± S.D	(Pa s) *x* ± S.D	(Pa s) *x* ± S.D	(1 h, 6 h, Tmax)
3	4	10	916 ± 21	689 ± 8	428 ± 16	12.43 ± 0.90 (1 h)
						62.51 ± 8.42 (6 h)
						Tmax: 78.70 ± 8.91 (8 h)
3	4	5	292 ± 7	227 ± 16	128 ± 4	11.05 ± 1.15 (1 h)
						61.97 ± 9.23 (6 h)
						Tmax: 84.93 ± 4.3 (7 h)
3	4	3	218 ± 5	186 ± 4	74 ± 13	9.01 ± 2.23 (1 h)
						62.31 ± 12.88 (6 h)
						Tmax: 68.17 ± 4.38 (8 h)
